# A phylogenomic approach to bacterial subspecies classification: proof of concept in *Mycobacterium abscessus*

**DOI:** 10.1186/1471-2164-14-879

**Published:** 2013-12-13

**Authors:** Joon Liang Tan, Tsung Fei Khang, Yun Fong Ngeow, Siew Woh Choo

**Affiliations:** 1 Department of Oral Biology and Biomedical Sciences, Faculty of Dentistry, University of Malaya, 50603, Kuala Lumpur, Malaysia; 2 Institute of Mathematical Sciences, Faculty of Science, University of Malaya, 50603, Kuala Lumpur, Malaysia; 3 Department of Medical Microbiology, Faculty of Medicine, University of Malaya, 50603, Kuala Lumpur, Malaysia; 4 Genome Informatics Research Laboratory, High Impact Research (HIR) Building, University of Malaya, 50603, Kuala Lumpur, Malaysia

## Abstract

**Background:**

*Mycobacterium abscessus* is a rapidly growing mycobacterium that is often associated with human infections. The taxonomy of this species has undergone several revisions and is still being debated. In this study, we sequenced the genomes of 12 *M. abscessus* strains and used phylogenomic analysis to perform subspecies classification.

**Results:**

A data mining approach was used to rank and select informative genes based on the relative entropy metric for the construction of a phylogenetic tree. The resulting tree topology was similar to that generated using the concatenation of five classical housekeeping genes: *rpoB*, *hsp65*, *secA*, *recA* and *sodA*. Additional support for the reliability of the subspecies classification came from the analysis of *erm41* and *ITS* gene sequences, single nucleotide polymorphisms (SNPs)-based classification and strain clustering demonstrated by a variable number tandem repeat (VNTR) assay and a multilocus sequence analysis (MLSA). We subsequently found that the concatenation of a minimal set of three median-ranked genes: DNA polymerase III subunit alpha (*polC*), 4-hydroxy-2-ketovalerate aldolase *(Hoa)* and cell division protein FtsZ (*ftsZ*), is sufficient to recover the same tree topology. PCR assays designed specifically for these genes showed that all three genes could be amplified in the reference strain of *M. abscessus* ATCC 19977^T^.

**Conclusion:**

This study provides proof of concept that whole-genome sequence-based data mining approach can provide confirmatory evidence of the phylogenetic informativeness of existing markers, as well as lead to the discovery of a more economical and informative set of markers that produces similar subspecies classification in *M. abscessus.* The systematic procedure used in this study to choose the informative minimal set of gene markers can potentially be applied to species or subspecies classification of other bacteria.

## Background

The rapidly growing mycobacteria (RGM), defined as mycobacteria that grow in culture media within seven days, are mostly environmental organisms, some of which have emerged as important opportunistic pathogens in humans, colonizers in the airway [1] or contaminants on surgical instruments [2]. More than a hundred species have so far been identified [3]. As different species and their subgroups are known to differ in their pathogenicity and susceptibility to antibiotics [4,5], their rapid and accurate identification is important for making therapeutic decisions in patient management. Unfortunately, although many taxonomic advances have been made in recent years, resulting in the creation of new species, subspecies and subgroups, the taxonomic status of some members is still unresolved.

*Mycobacterium abscessus* is generally regarded as the most important RGM associated with human infections that range from localized cutaneous inflammation to rare but serious disseminated sepsis [6,7]. It is responsible for more than 80% of the chronic lung diseases caused by RGM, some of which require surgical resection of the infected lung for complete resolution of symptoms [8]. This organism shows many similarities with another RGM, *M. chelonae,* and was once classified as *M. chelonae* subspecies *abscessus *[9]. With DNA-DNA hybridization studies, however, it became recognized as a distinct species [10,11]. Subsequently, the species was divided into three subspecies: *M. abscessus* sensu stricto, *M. massiliense* and *M. bolletii *[12-14] but the most recent classification describes just two subspecies which are *M. abscessus* subspecies *abscessus* (formerly *M. abscessus* sensu stricto) and *M. abscessus* subspecies *bolletii* that includes the previous two subspecies of *M. bolletii* and *M. massiliense *[15]. However, in many phylogenetic and phylogenomic studies, *M. abscessus* is clearly separated into three subgroups [16-18]. Clinical and population studies have also indicated notable differences among the three subspecies [16,17,19]. Hence, in this paper, we use the former three subspecies classification for ease of reference to earlier publications.

Traditionally, species identification for RGM is based on biological and biochemical tests such as pigment production, 3-day arylsulfatase reaction, nitrate reduction, iron uptake, and tolerance to 5% NaCl. Antibiotic susceptibility has also been used to assist in species or subspecies classification. For instance, polymyxin B inhibits *M. fortuitum* but not *M. abscessus *[20], while in the *M. abscessus* group, *M. massiliense* can be differentiated from the other two subspecies by a truncated erythromycin ribosome methyltransferase (*erm 41*) gene associated with susceptibility to macrolide antibiotics [19]. Although easy to use, the accuracy of these tests can be easily affected by the phenomenon of horizontal gene transfer (HGT) and differential gene expression. Therefore, phenotypic tests have largely been replaced by modern genotypic methods.

The PCR-Restriction Enzyme analysis (PRA) is one of the most commonly used genotypic methods. Direct sequencing of PCR amplified products based on the polymorphism of housekeeping genes has also become available in many diagnostic laboratories. In both methods, the *hsp65* gene, which is highly conserved within species, is frequently used. Most of the RGM currently known can be identified from RE patterns generated from a 439 bp variable portion of the gene [21,22]. In PCR-sequencing, the *hsp65* gene shows more variability among RGM species than the 16S rRNA gene, the universally conserved gene chosen for the phylogenetic analysis of prokaryotes. Thus, it is better than the latter for the identification of closely-related mycobacterial species [23]. Other housekeeping genes that have been used in RGM studies, albeit at lower frequency, include the internal transcribed spacer (*ITS*) of the 16S-23S rRNA gene [24], the *rpoB *[25], *sodA *[26], *secA *[16]*. recA *[27] and *gyrB *[28] genes. Commercially available reverse line probe hybridization assays (INNO-LIPA Mycobacteria, Innogenetics, Ghent, Belgium; GenoType Mycobacterium CM/AS, Hain Lifescience Gmbh, Germany) are increasingly being used as convenient molecular tools for rapid subspecies identification in diagnostic laboratories.

The conventional phylogenetic approach uses single or limited numbers of genes to infer phylogenetic relationships among the taxa of interest. Classification of taxa can then be done on the basis of the topology of the inferred phylogenetic tree. A potential drawback of this method is that the optimality of phylogenetic signal in the genes used, as quantified using some suitable metric, is largely unknown because there is no systematic procedure to find suitable candidates from a population of genes. The recent introduction of next-generation sequencing has made it possible to use whole- genome sequence information for subspecies classification via phylogenomic clustering. An example is the phylogenomic reconstruction of lactic acid bacteria by Zhang *et al*. [29]. The purpose of this study is to test the phylogenetic informativeness of five classical housekeeping genes: *rpoB*, *hsp65*, *secA*, *recA* and *sodA* that are commonly used in *M. abscessus* subspecies classification. Concurrently we wish to propose a phylogenomic approach to identify, to the subspecies level, 12 *M. abscessus* strains isolated from clinical samples, using a smaller set of genes that is at least as informative as the five classical genes for the purpose of inferring a high-confidence tree topology.

## Results

### Genome assembly, annotation and identification of orthologs

The draft genomes of our 12 *M. abscessus* strains, assembled using CLC Genomics Workbench and annotated using RAST, showed genome sizes ranging from 4,802,413 bp to 5,488,620 bp with an average of 5,054,711 bp. The number of putative Coding Sequences (CDS) ranged from 4,709 to 5560 (Additional file 1: Table S1). With the use of BLASTClust on the putative CDS from all genomes including those of the three reference strains and the three outgroup species, 210 orthologous genes were identified.

### Single and multiple gene-based subspecies classification

**Figure 1:**
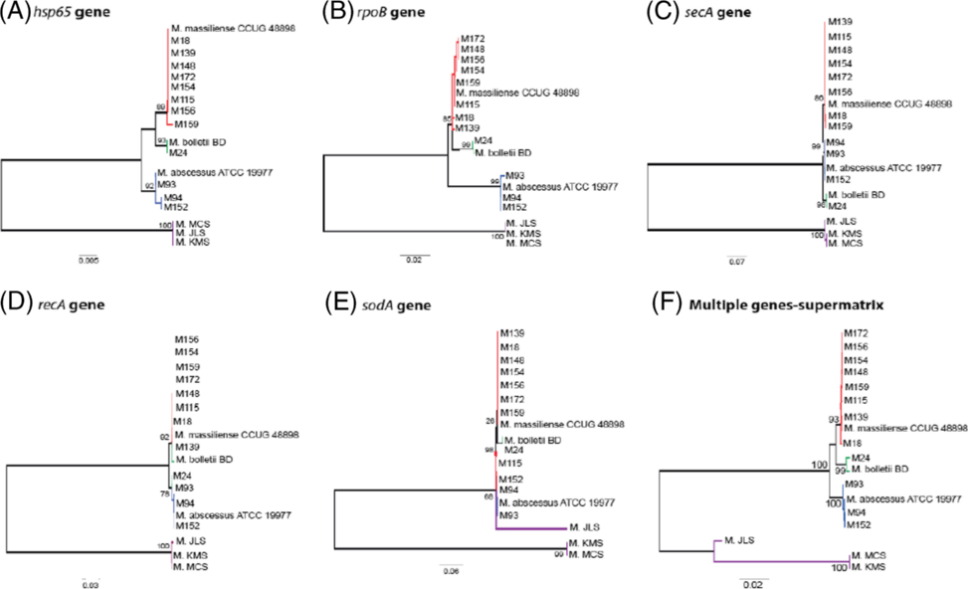
**Phylogenetic inference using classical genes.** The trees were constructed using the maximum likelihood method, with 1000 bootstrap replicates. Only bootstrap values at important branches are shown. All the trees were inferred based on Tamura 3-parameter model with complete deletion option but at different substitution rates. **(A)***hsp65*-based tree (Uniform rate), **(B)***rpoB*-based tree (Gamma rate), **(C)***secA*-based tree (Gamma rate), **(D)***recA*-based tree (Gamma rate), **(E)***sodA*-based tree (Uniform rate), **(F)** supermatrix tree (Gamma rate).

On comparing the phylogenetic trees constructed using the five common marker genes for *M. abscessus* subspecies classification, the single gene- based trees inferred using *rpoB, hsp65* and *secA* genes showed clear and consistent classification of each isolate into subspecies (Figure 1A-C). The *recA* gene, however, gave a slightly different classification (Figure 1D). For instance, the two reference strains, *M. bolletii* BD^T^ and *M. massiliense* CCUG 48898^T^*w*ere grouped together and strain M24 was grouped with *M. abscessus* ATCC 19977^T^, instead of *M. bolletii* BD^T^ as in the *rpoB-, hsp65-* and *secA-*derived trees. The *sodA* -based tree was also different in that strain M115 was identified as *M. abscessus* sensu stricto instead of *M. massiliense* as observed in the other trees (Figure 1E). These results indicate that *sodA* and *recA* marker genes, which are commonly used for mycobacterial classification, may not be suitable for *M. abscessus* subspecies identification.

The supermatrix tree obtained from the concatenation of the five genes has also revealed three clear subspecies grouping supported by strong bootstrap values of 93% for *M. massiliense*, 99% for *M. bolletii* and 100% for *M. abscessus* sensu stricto (Figure 1F). The classification for our 12 strains was similar to those obtained with the *rpoB*, *hsp65* and *secA* genes.

### Phylogenomic approach to subspecies classification

**Figure 2:**
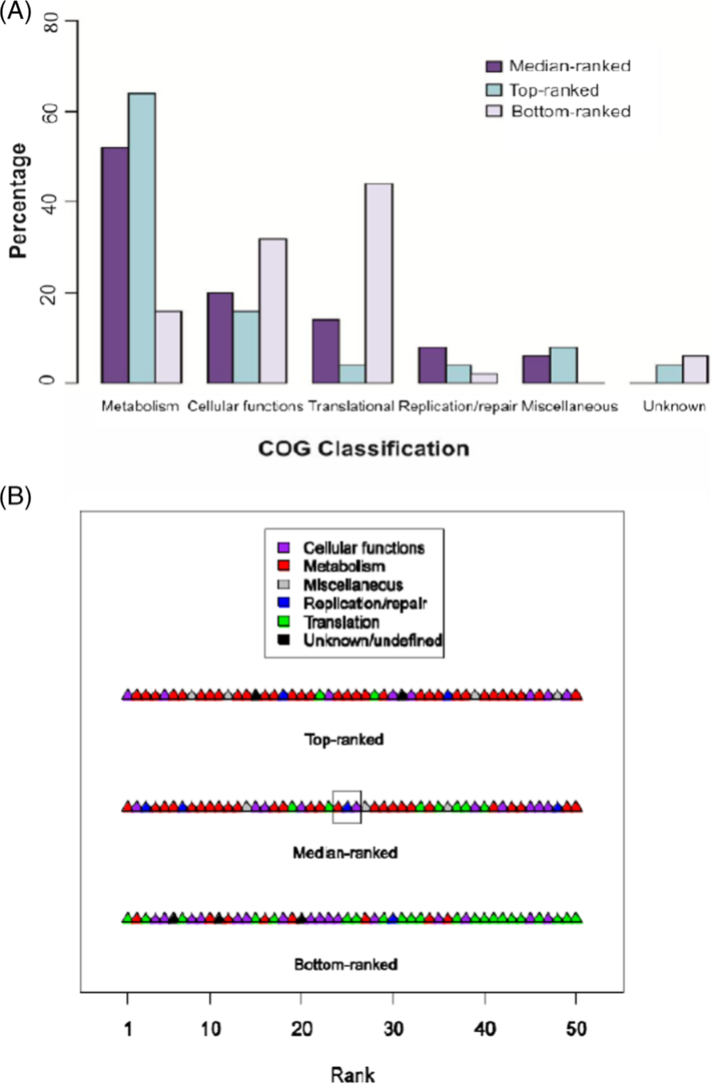
**Functional analysis of orthologs. (A)** COG classification composition of genes in the top, median and bottom-ranked genes. **(B)** COG classification of genes in the top, median and bottom-ranked genes. The highest relative entropy is found in the left-most member of the top-ranked genes; the lowest entropy is found in the right-most member of the bottom-ranked genes. The set of minimal genes capable of reconstructing the clustering topology as the one obtained using 50 median-ranked genes is boxed.

The availability of whole-genome sequences enabled us to systematically evaluate the phylogenetic informativeness of a large pool of candidate genes and then select optimal ones for phylogenetic tree construction. For this evaluation, we used the same procedure to predict the Open Reading Frames (ORFs) in the 12 genomes sequenced in this study, as well as the genomes of the reference and outgroup species. On aligning the sequences of the 210 orthologous genes identified with BLASTClust, we found the MSA produced using MAFFT [30] to be more robust than that produced using MUSCLE [31]. Subsequently, we chose the MAFFT-aligned MSA for downstream analysis. The phylogenetic informativeness of each gene was assessed and ranked based on calculated entropy values as described in the Methods section. By using this systematic approach, we selected 50 median-ranked genes which would be the most phylogenetically optimal for the purpose of subspecies phylogeny inference. The functional analysis of these 50 median-ranked genes, as well as the 50 top-ranked and 50 bottom ranked genes showed that the majority of the bottom-ranked genes are involved in translational processes, whereas those of the top-ranked genes are mostly involved in enzyme metabolism (Figure 2; Additional file 1: Table S2–S4). These results indicate that the ranking obtained using the relative entropy metric appears to be biologically reasonable.

**Figure 3:**
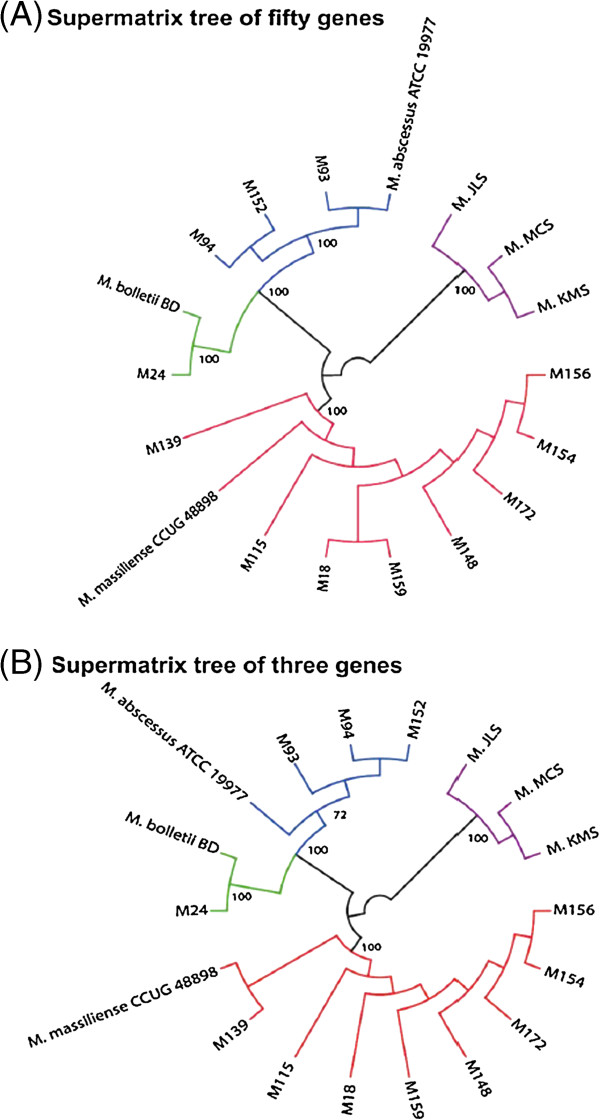
**Supermatrix phylogenomic trees (General Time Reversible model with Gamma substitution rate, include all codon positions, complete deletion option). (A)** Fifty-gene tree. **(B)** Three-gene tree. Phylogenetic trees generated from the concatenation of the selected genes, constructed with the maximum likelihood method with boostrap values at important branches (1,000 bootstrap replicates). As shown in **(B)**, the concatenation of three genes is sufficient to construct a phylogenetic tree showing subspecies classification similar to that in the supermatrix tree of fifty genes. As anticipated, of the 12 *M. abscessus* isolates analysed, only M24 is classified as *M. bolletii*, supported with 100% bootstrap support. M93, M94 and M152 are classified as *M. abscessus* sensu stricto and others as *M. massiliense*.

In the phylogenomic tree constructed based on the selected 50 median-ranked genes (Figure 3A), the classification of isolates inferred agreed with the one inferred using the five concatenated classical housekeeping genes. Strains M93 to M94 were clustered with *M. abscessus* sensu stricto; M24 with *M. bolletii* and the others with *M. massiliense*.

To find a minimal set of genes capable of producing the same subspecies classification as that obtained using the 50 median-ranked genes, we iteratively removed genes from the left and right ends of the list of median-ranked genes, and used the remaining genes to construct the phylogenetic tree. The process was terminated when the classification implied by the tree topology changed. Thus, we found that the minimum number of median-ranked genes needed for recovering similar subspecies classification (Figure 3B) attained using the 50 median-ranked genes was three. These three genes were DNA polymerase III subunit alpha (*polC*; Accession: YP_001703430.1), 4-hydroxy-2-ketovalerate aldolase (*Hoa*; Accession: YP_001701378.1) and cell division protein FtsZ (*ftsZ*; Accession: HQ662067.1). We confirmed the amplifiability of informative fragments from these three genes in *M. abscessus* ATCC 19977^T^ (Additional file 1: Figure S1; Additional file 1: Table S5).

### Evidence to support the subspecies classification by phylogenomics analysis

#### Examination of *erm41* and 16S-23S rRNA *ITS* genes

**Figure 4:**
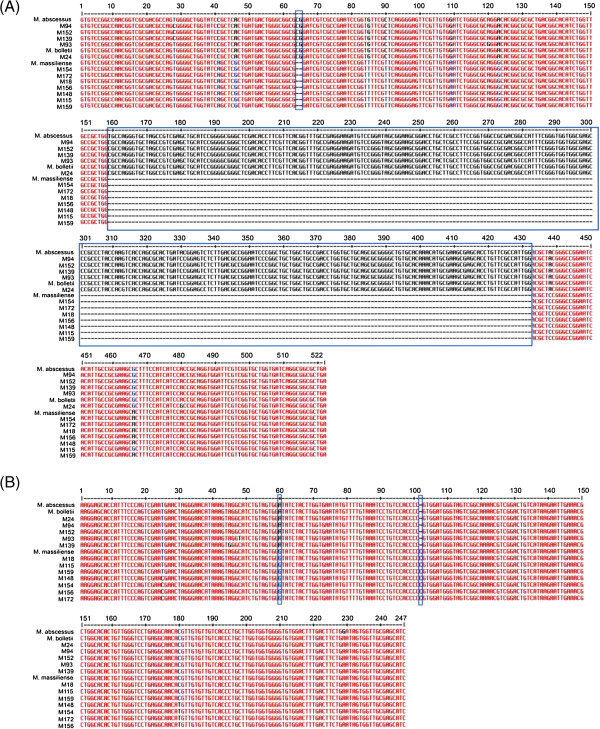
**Multiple alignments of *erm41 *and 16S-23S rRNA *ITS *gene sequences. (A)** Multiple alignment of *erm41* sequences revealed deletions at positions 64^th^ and 65^th^, in addition to a large deletion (boxed in blue) in all *M. massiliense* strains, except M139. **(B)** Multiple alignment of 16S-23S rRNA *ITS* gene sequences showed A to G substitution at position 60^th^ and insertion of C at position 102^nd^ (boxed in blue) in all *M. massiliense* strains except for M139.

Previous studies have shown that the *erm41* and *the* 16S-23S rRNA *ITS* genes could be used to differentiate the subspecies of *M. abscessus *[12,19]. We examined the sequences of these genes in all 12 strains and found seven strains (M156, M148, M154, M172, M152, M115 and M18) having deletions at the 64^th^ and 65^th^ positions and a large deletion from position 159^th^ to 432^nd^ in the *erm41* gene (Figure 4A), as well as an A to G substitution at position 60^th^ and insertion of C at position 102^nd^ in the *ITS* gene (Figure 4B). Since these features are typically found in *M. massiliense*, their presence in the seven strains further supported the classification of these strains as *M. massiliense,* as observed in the supermatrix analyses. However, M139 which was classified as *M. massiliense* in all the single gene-based and multiple gene-based approaches did not have these expected features.

#### Classification of other *M. abscessus* strains from public database

**Figure 5:**
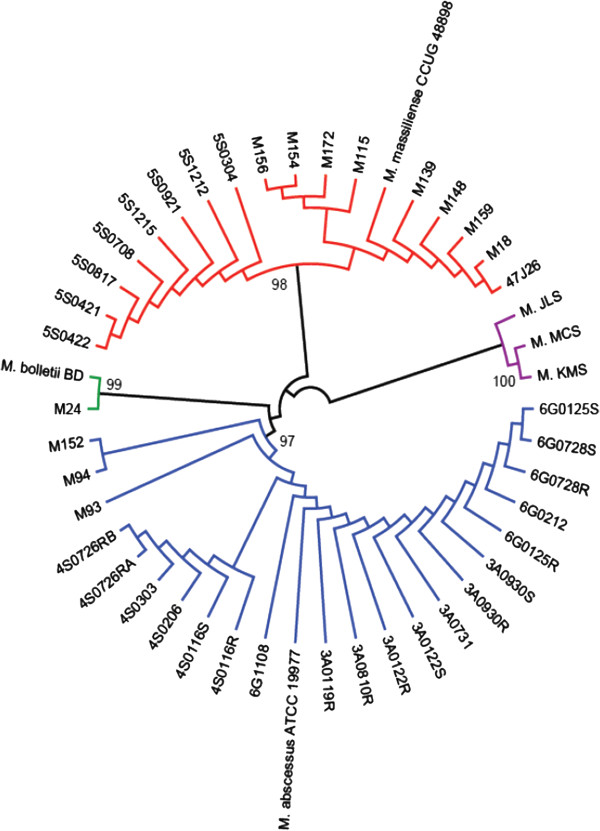
**Phylogenetic tree based on three marker genes, for 43** ***M. abscessus *strains and three outgroups (Tamura 3-parameter model with Gamma substitution rate, include all codon positions, complete deletion option).** Of the 28 additional *M. abscessus* strains, this phylogenomics analysis revealed 19 strains as *M. abscessus* sensu stricto and 9 strains as *M. massiliense*, supported by 97% to 98% bootstrap support.

At the end of our study, many *M. abscessus* genome sequences have been deposited in NCBI Genbank database. To further test the reliability of the three selected gene markers, we used them to classify 43 *M. abscessus* genomes (12 from our laboratory; 28 deposited by other researchers in the NCBI Genbank database; three reference) and three outgroup genomes (Additional file 1: Table S6). The phylogenetic tree constructed using the minimal set of three genes clearly showed three major groups corresponding to the three *M. abscessus* subspecies (Figure 5). For example, the strains from 6G0125S to 4S0726RB were grouped into the *M. abscessus* sensu stricto cluster, whereas the strains from 47J26 to 5S0422 were likely to be *M. massiliense*. None of the additional 28 strains were identified as *M. bolletii*. This classification was in accordance with and supported by the analysis of *hsp65* and *rpoB* gene sequences (Additional file 1: Figure S2). The addition of 28 strains into this study did not change the classification of the 12 strains from our initial analysis. These results again support our belief that the three selected genes are reliable markers for the classification of *M. abscessus* subspecies.

#### Other supporting evidence

Recently, Wong *et al*. [32] showed that a variable-number tandem-repeat (VNTR) typing assay for the *M. abscessus* species seemed to give good differentiation of *M. abscessus* strains. In their study, both the VNTR assay and a multilocus sequence analysis assay (MLSA) based on seven housekeeping genes (*argH*, *cya*, *glpK*, *gnd*, *murC*, *pta*, and *purH*) yielded subspecies clustering that is concordant (with the exception of M139) with the one inferred using the present phylogenomics approach.

In addition, the availability of whole genome sequences of the 12 *M. abscessus* strains allowed us to proceed with the reconstruction of a phylogenetic tree using single nucleotide polymorphisms (SNPs) in the genomic regions that are conserved across all strains (Additional file 1: Figure S3). The resulting SNP-based tree showed that the classification of the 12 *M. abscessus* strains is similar to the classification obtained from the phylogenomics tree generated using our systemic data mining approach.

**Figure 6:**
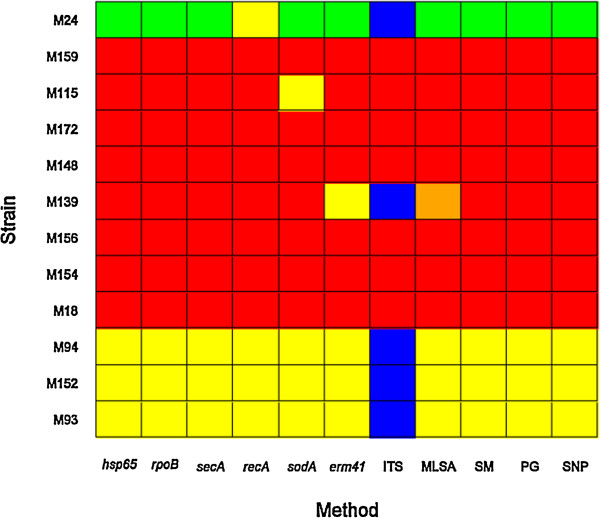
**Classification of strains according to different methods.** Color legend: Yellow for *M. abscessus* sensu stricto; red for *M. massiliense;* green for *M. bolletii*; blue for *M. abscessus* sensu stricto*/M. boletti*; orange for *M. abscessus* sensu stricto*/M. massiliense*. Abbreviation: SM: supermatrix; PG: phylogenomics; SNP: single-nucleotide polymorphism.

The classification results of the 12 sequenced genomes using different approaches are summarized in Figure 6.

## Discussion

Using a proposed phylogenomic approach, we have successfully classified into subspecies, 12 sequenced genomes of *M. abscessus* strains isolated from clinical samples. The primary value of this approach is the use of a set of objectively selected genes for constructing a high confidence tree topology that can serve as a benchmark. Subsequently, the classification implicit in the topology of the inferred tree can be used to guide the selection of a minimal gene set for practical, routine use. Existing methods (multiple-genes, MLSA) are not able to produce such benchmarks, and their resulting classification accuracy may be largely a matter of luck rather than science. Corroboration from the analysis of whole-genome SNP data further convinces us of the usefulness of the phylogenomic approach to systematically discover a minimal gene set for subspecies classification, at least in *M. abscessus*.

Our phylogenomic approach uncovered a set of genes detected using median–ranking that supports the subspecies classification inferred using the concatenation of the five classical genes. The set of median-ranked genes has good coverage of all important functional classes compared to the top and bottom-ranked genes, so using their concatenation for phylogenetic inference potentially avoids systematic biases that can be caused by having a concentration of genes in the ranked list for some particular functional class. Further refinement resulted in a new, three-member gene set that preserves the similar classification. The three selected genes are biologically interesting and come from three different functional classes. The KEGG database [33] shows that DNA polymerase III subunit alpha (*polC*) functions in DNA replication; 4-hydroxy-2-ketovalerate aldolase is involved in phenylalanine metabolism, degradation of benzoate, dioxin and xylene; and cell division protein gene *ftsZ* produces the FtsZ GTP-binding cell division protein which is important for the cytoskeleton formation during cell wall synthesis. A brief survey of available literature shows that *polC* has been used as a phylogenetic marker in *Bacillus subtilis* subspecies classification [34] as well as lactic acid bacteria phylogeny [29]. In *Wolbachia* phylogenetic studies, *ftsZ* has been used in conjunction with other genes [35,36].

In this study, we also assessed a set of genes commonly used by other workers for the subspecies classification of *M. abscessus* (*rpoB*, *hsp65*, *recA*, *secA, sodA, erm41* and *ITS*) and found inconsistent classification for certain strains with *recA, sodA, erm41* and *ITS* genes. It is well-known that the single gene-based approach is sensitive to the evolutionary history of the gene and not necessarily of the species, and may be limited by the lack of sufficient variation in a single gene sequence [37-39]. A multiple gene-based approach, if the genes are carefully chosen, may amplify the individual phylogenetic signals present in the genes and lead to an accurate inference of phylogenetic tree for subspecies classification [40,41]. This is seen in the superior robustness of our supermatrix trees whether they are based on the concatenation of five classical genes, core genome SNPs or our three gene markers.

Of the 12 strains we isolated, M139 had the most ambiguous taxonomic identity. It was classified as *M. massiliense* in the current study and also in two recent publications [17,18] using our *M. abscessus* genome sequences for comparative analyses. However, it had features of *M. abscessus* sensu stricto in the *erm41* and *ITS* genes and was shown to cluster with *M. abscessus* in the MLS and VNTR assays reported by Wong *et al. *[32]. Additionally, it was identified as *M. abscessus* subsp. *abscessus* in the MALDI Biotyper system (Bruker, Germany) that uses the MALDI-TOF (matrix-assisted laser desorption ionization-time of flight) mass spectrometry to identify organisms by their molecular (protein) fingerprints. The *erm41* gene in M139 is intact but the minimum inhibitory concentration (MIC) of clarithromycin obtained with the Epsilometer test (bio Merieux, France) was 0.094 mg/l even after prolonged incubation up to 14 days. This high level of *in vitro* susceptibility is more often seen among *M. massiliense* than *M. abscessus* or *M. bolletii* strains. In M139, however, this low MIC can be attributed to the C28 polymorphism in the *erm41* gene which has been reported to prevent the expression of the inducible clarithromycin resistance conferred by an intact *erm41* gene [42].

The apparent incongruities in the subspecies classification of M139 could be because previous studies used insufficient numbers of strains to describe “typical” features associated with the different *M. abscessus* subspecies. More extensive sampling of *M. abscessus* populations from different geographical locations and clinical settings would help to resolve current taxonomic uncertainties.

## Conclusion

Whole-genome sequencing of medically important bacterial strains opens up new possibility for accurate subspecies classification via a phylogenomic approach. We have shown how a large pool of orthologs can be identified bioinformatically from the genomes of 12 *M. abscessus* isolates, assembled from fragments generated using Illumina shotgun sequencing. We then introduced a method to rank the phylogenetic informativeness of the identified orthologs using the relative entropy metric. Based on the set of 50 median-ranked genes, a benchmark phylogeny was obtained, and we used its topology to infer subspecies classification. We made the method practical by discovering a three-member minimal gene set that could return the same subspecies classification obtained using 50 median-ranked genes. The workflow here provides an objective means for the development of molecular-based classification method that was not possible with previous methods such as single gene, arbitrary multiple gene approaches, VNTR as well as MLSA. Our three gene set is at least as informative as classical markers commonly used by other researchers. Its applicability can be further evaluated by testing it on a larger population of strains, from different clinical settings and geographical locations.

## Methods

### Bacterial strains

The 12 *M. abscessus* strains used in this study were collected between July 2009 and June 2011 from the Clinical Microbiology Laboratory of University of Malaya Medical Centre (UMMC), Kuala Lumpur, Malaysia. With the exception of one strain from a lymph node, all other strains were from sputum or bronchoalveolar fluid of patients presenting with respiratory infections. In the UMMC laboratory, clinical specimens from patients suspected to have tuberculosis are routinely cultured in the BACTEC MGIT_960_ liquid culture system (Becton-Dickinson). Positive cultures are examined microscopically and subcultured on Lowenstein-Jensen (LJ) slants. Acid-fast colonies on LJ slants are then identified using a variety of tests. The strains used in this study were identified as *M. abscessus* by their rapid growth (within seven days of incubation) in the MGIT_960_ system, non-pigmented acid-fast colonies on LJ slants, typical restriction pattern in a PCR-restriction fragment length polymorphism analysis [21] and a positive identification in a reverse line probe hybridization assay (GenoType Mycobacterium CM/AS; Hain Lifescience GmbH, Germany).

All isolates were kept in Middlebrook 7H9 broth with 15% glycerol, at -80°C, until required for further testing.

For whole-genome sequencing, archived strains were retrieved, rendered non-nominal and subcultured on LJ slants. This study involved only genomic analysis of routine isolates and, with the exception of specimen type and geographical origin, none of the data used could expose patient identity. As such, we considered it unnecessary to apply for ethical approval by the University's Medical Ethics Committee Standard Operating Procedures (http://www.ummc.edu.my/view/content.php?ID=56).

The genome information of the reference strains was extracted from the NCBI Genbank [43] database. The reference strains used for comparison are given in Additional file 1: Table S6.

### DNA extraction & isolate validation

Pure cultures on LJ slants were harvested by flooding each slant with 3 ml of phosphate buffered saline (PBS, Ph 7.4) followed by gentle scraping of the agar surface with a glass Pasteur pipette. The suspension was then collected into a 15 mL centrifuge tube and vortex mixed for 5 minutes. For DNA extraction, 200 μL of suspension was processed with the ZR Fungal/Bacterial DNA MiniPrepTM (Zymo Research, USA) according to the protocol provided by the manufacturer. DNA concentration and purity were measured on the NanoDrop2000 spectrophotometer (Thermo Scientific). To validate the identity of the 12 *M. abscessus* isolates, we used PCR to amplify their *hsp65* genes for Sanger sequencing, and then used BLAST to query the sequences to known *Mycobacterium* species/subspecies gene sequences stored in a web-accessible database of *hsp65* locus sequences [44]. All 12 strains achieved almost perfect or perfect (98% to 100%) similarity to *M. abscessus*.

### Whole-genome sequencing, annotation and identification of orthologs

The genomes of the 12 isolates were shotgun-sequenced using Illumina Genome Analyzer 2X technology. The raw sequencing reads were trimmed at a threshold of 0.01 and the sequences obtained were assembled *de novo* using CLC Genomics Workbench version 4.9 (CLC bio, Denmark). All target genes for analysis were extracted from the assembled genomes of the 12 *M. abscessus* strains. The gene sequences for the reference *M. abscesuss* subspecies were obtained from the NCBI database. We used BLAST [45] to locate the boundaries of the genes in the 12 assembled draft genomes.

We annotated the genomes of strains included in this analysis using Rapid Annotation running on Subsystem Technology (RAST) automated pipeline [46]. The orthologs among the putative open reading frames (ORFs) were identified using BLASTClust in a standalone BLAST program [47].

### Phylogenetic classification using classical markers

The pan-bacterial 16S rRNA gene, which is generally considered the best target for bacterial identification and phylogenetic analysis, was not used in this study as it has been reported to lack discriminatory power for *M. abscessus* subspecies [48]. Instead, five other genes that have been used by other workers were chosen for phylogenetic analysis, namely, the *rpoB* (711 bp) gene encoding for the *β*-subunit of RNA polymerase enzyme responsible for RNA synthesis [49]; the *hsp65* (603 bp) gene for the 65 kDa heat shock protein that is involved in intracellular protein folding, assembly and transport [50]; the *sodA* (539 bp) gene encoding superoxide dismutase, a metalloenzyme that contributes to defence against oxidative stress in mycobacteria [51]; the *recA* (1041 bp) recombinase gene related to DNA repair [52] and the *secA* (700 bp) gene that is involved with pre-protein transport in the bacterial cell [53]*.*

Each of the five housekeeping gene sequences was aligned using ClustalX from the European Bioinformatics Institute (EBI) online server to study similarities and variable sites among the subspecies [54]. We then used MEGA version 5.10 [55] to select the best DNA substitution model for each multiple sequence alignment (MSA), followed by maximum likelihood estimation of trees. In addition, we concatenated all five genes, and similarly used the MSA of the latter to infer maximum likelihood trees (1,000 bootstrap replications).

Two additional genes, *erm41* and 16S-23S rRNA *ITS* with known features for subspecies identification were used to further support the classification results. *M. massiliense* can be recognized by the presence of a truncated *erm41* gene [19] as well as an A to G substitution at the 60th position and a C insertion at the 102nd position of the 16S-23S rRNA *ITS *[12].

### Phylogenomic classification using whole-genome data

Not all genes have the same phylogenetic signal [56]. The main advantage of the phylogenomic approach is that it allows the generation of a large population of orthologs in the taxa of interest so that an objective assessment of their phylogenetic informativeness can be made. These genes can be ranked accordingly based on some metric that is entropy-dependent for the purpose of quantifying phylogenetic informativeness. Given a list of genes ranked from high to low entropy, we propose that a subset of genes in the neighbourhood of the median of this ranked list would be the most phylogenetically optimal for the purpose of subspecies phylogeny inference. If this method works, a simple functional categorization of genes in the top, median and bottom-ranked genes should roughly show the following pattern: dominance of genes involved in the highly conserved translational process in the bottom-ranked genes, and dominance of genes involved in metabolism in the top-ranked genes. The subsequent phylogenetic tree constructed using the median-ranked genes would then constitute a high confidence phylogeny.

To prepare the genes for ranking, we performed multiple sequence alignment (MSA) for the orthologs identified. Next, we removed all MSA with alignment gaps greater than 5% of the size of the alignment block. To reduce the chances of using genes that are not robust to choice of the MSA algorithm, we compared the results of MUSCLE and MAFFT. We chose these two algorithms because of positive evaluations with respect to their accuracy and speed performance [57]. To exclude co-optimality of MSA as a potential source of alignment error, we further performed the Heads or Tails (HoT) analysis [58] on the identified orthologs. In HoT analysis, an MSA using reversed sequences was first built; this MSA was then reversed and then compared against the MSA built using the original, unreversed sequences. Only genes that show identical MSA after an HoT analysis were subsequently used for downstream analysis.

To quantify the phylogenetic signal of the *i*th gene (*g*_*i*_) gene, we used the relative entropy metric for a gene’s MSA:

$$H\left(g_{i}\right)=\sum_{x=1}^{s}\sum_{y=1}^{L}p_{\mathrm{xy}}\left(\mathrm{nt}\right)\log\frac{p_{\mathrm{xy}}\left(\mathrm{nt}\right)}{p_{x}\left(\mathrm{nt}\right)p_{y}\left(\mathrm{nt}\right)},$$

where *s* is the number of sequences, *L* is the length of the MSA, *p*_*xy*_*(nt)* is the relative frequency of state *nt* (gaps are treated as a fifth character) in the entire MSA at row *x* and column *y*, with *nt ∈ {A, G, C, T,-}*, *p*_*x*_*(nt)* is the row marginal frequency for state *nt*, and *p*_*y*_*(nt)* is the column marginal frequency for state *nt*. This metric measures the divergence between the nucleotide frequencies in the entire MSA from the nucleotide frequencies in the rows and columns of the MSA. An MSA with noiseless columns would yield a zero value since the nucleotide frequencies in the columns would be degenerate, and those in the rows would be the same as the nucleotide frequencies in the MSA. For MSA with noisy columns, *H* increases. All computation was done using an in-house R script [59] (available upon request).

Fifty genes - 25 to the left and right (in alternating order) of the list of median-ranked genes, were concatenated and used to reconstruct the phylogenetic tree of the 12 isolate strains. Taking the clustering of strains based on the 50 median-ranked genes as the gold standard, we subsequently deduced the set of minimum median-ranked genes needed to recover the same clustering by iteratively reducing the number of median-ranked genes used and checking the resulting change in clustering topology. Candidate markers in this minimum set were then tested for their amplifiability using a standard PCR protocol to establish their practical usefulness as new, objectively inferred phylogenetic markers.

## Competing interests

The authors have declared that no competing interests exist.

## Authors’ contributions

Conceived and designed the experiments: all authors. Performed the wet laboratory experiments and contributed reagents/materials: YFN. Analyzed the data: JLT, SWC and TFK. Wrote the paper: all authors. All authors read and approved the final manuscript.

## Supplementary Material

Additional file 1 Supplementary file. Click here for file
