# Supplementary material for: A phylogenomic approach to bacterial subspecies classification: proof of concept in Mycobacterium abscessus
Source: BMC Genomics. 2013 Dec 13;14:879. doi: 10.1186/1471-2164-14-879 (PMC3878664; doi:10.1186/1471-2164-14-879)
Supplement: Additional file 1 — Supplementary file. [file 1471-2164-14-879-S1.pdf]

A Phylogenomic Approach to Bacterial Subspecies Classification: Proof of Concept in *Mycobacterium abscessus*

Joon Liang Tan[3,4], Tsung Fei Khang[2*], Yun Fong Ngeow[3], Siew Woh Choo[1,4]*

[1]Department of Oral Biology and Biomedical Sciences, Faculty of Dentistry, University of Malaya, 50603 Kuala Lumpur, Malaysia

[2]Institute of Mathematical Sciences, Faculty of Science, University of Malaya, 50603 Kuala Lumpur, Malaysia

[3]Department of Medical Microbiology, Faculty of Medicine, University of Malaya, 50603 Kuala Lumpur, Malaysia

[4]Genome Informatics Research Laboratory, High Impact Research (HIR) Building, University of Malaya, 50603 Kuala Lumpur, Malaysia

***=Corresponding authors:**

Siew Woh Choo

Contact: lchoo@um.edu.my

Tsung Fei Khang

Contact: tfkhang@um.edu.my

Supplementary Table 1. Summary of genome assembly and annotation

Strain	Source	Genome Size (bp)	Patients' Nationality	No. CDS
M93	Sputum	5,080,377	Malaysian	4,970
M94	Sputum	5,101,275	Malaysian	5,146
M152	Sputum	4,915,341	Malaysian	4,833
M115	Sputum	4,978,401	Malaysian	4,947
M139	Sputum	5,046,765	Nepalese working in Malaysia	4,983
M148	Sputum	5,171,956	Malaysia	5,363
M172	Sputum	5,207,258	Myanmar working in Malaysia	5,244
M156	Sputum	5,025,652	Malaysian	5,039
M24	Bronchoaveolar lavage	5,488,620	Malaysian	5,560
M159	Bronchoaveolar lavage	4,936,811	Malaysian	4,864
M154	Bronchoaveolar lavage	4,802,413	Malaysian	4,709
M18	Lymph node	4,889,208	Malaysian	4,850

Supplementary Table 2. Functional classification of the 50 median-ranked genes.

**Gene**	**Functional Classification**
CysN/CysC bifunctional enzyme	Metabolism
PhoH-like protein	Cellular Functions
excinuclease ABC subunit A	Replication/repair
phosphogluconate dehydrogenase	Metabolism
glutamate decarboxylase GadB	Metabolism
acyl-CoA dehydrogenase FadE	Metabolism
chromosomal replication initiation protein	Replication/repair
porphobilinogen deaminase	Metabolism
acetolactate synthase 1 catalytic subunit	Metabolism
putative ferredoxin-dependent glutamate synthase	Metabolism
hypothetical protein MAB 0584	Metabolism
acetyl-CoA acetyltransferase	Metabolism
acetaldehyde dehydrogenase	Metabolism
macrolide ABC transporter ATP-binding protein	Miscellaneous
succinate dehydrogenase flavoprotein subunit	Cellular Functions
oxoglutarate ferredoxin oxidoreductase subunit beta	Cellular Functions
pobable propionyl-CoA carboxylase subunit beta AccD2	Metabolism
bifunctional 3,4-dihydroxy-2-butanone 4-phosphate synthase/GTP cyclohydrolase II protein	Metabolism
aspartyl-tRNA synthetase	Translation
zinc-dependent alcohol dehydrogenase AdhE2	Cellular Functions
carbamoyl phosphate synthase large subunit	Metabolism
inosine 5'-monophosphate dehydrogenase	Metabolism
30S ribosomal protein S17	Translation
4-hydroxy-2-ketovalerate aldolase	Metabolism
DNA polymerase III subunit alpha	Replication/repair
cell division protein FtsZ	Cellular Functions
Rieske family iron-sulfur cluster-binding protein	Miscellaneous
acyl-CoA dehydrogenase FadE	Metabolism
methylmalonyl-CoA mutase	Metabolism
argininosuccinate synthase	Metabolism
aconitate hydratase ACN	Metabolism
4-hydroxy-3-methylbut-2-enyl diphosphate reductase	Metabolism
30S ribosomal protein S5	Translation
3-deoxy-D-arabino-heptulosonate 7-phosphate synthase AroG	Metabolism
30S ribosomal protein S9	Translation
hypothetical protein MAB 1619	Miscellaneous
glycyl-tRNA synthetase	Translation
30S ribosomal protein S3	Translation
dihydrolipoamide dehydrogenase	Cellular Functions
DNA helicase	Translation
biotin synthase	Metabolism

ABC transporter ATP-binding protein	Cellular Functions
sulfate adenylyltransferase subunit 2	Metabolism
nucleoside diphosphate kinase	Metabolism
phosphoenolpyruvate carboxykinase	Cellular Functions
electron transfer flavoprotein subunit beta FixA	Cellular Functions
succinyl-CoA synthetase subunit beta	Cellular Functions
DNA gyrase subunit B	Replication/repair
myo-inositol-1-phosphate synthase	Metabolism
serine hydroxymethyltransferase	Metabolism

Supplementary Table 3. Functional classification of the 50 top-ranked genes.

**Gene**	**Functional Classification**
ABC transporter ATP-binding protein	Cellular Functions
beta-lactamase-like	Metabolism
MCE-family protein	Metabolism
ABC transporter ATP-binding protein	Metabolism
hypothetical protein MAB 2234c	Cellular Functions
phosphoribosylglycinamide formyltransferase	Metabolism
NADH dehydrogenase subunit L	Metabolism
serine/threonine-protein kinase PknB	Miscellaneous
citrate lyase beta chain	Metabolism
N-succinyldiaminopimelate aminotransferase	Metabolism
acyl-CoA dehydrogenase FadE	Metabolism
hypothetical protein MAB 2411c	Miscellaneous
uroporphyrinogen decarboxylase HemE	Metabolism
bacterioferritin BfrB	Metabolism
arabinosyltransferase C	Unknown/undefined
putative biotin sulfoxide reductase BisC	Metabolism
branched chain amino acid ABC transporter	Metabolism
ATP-dependent DNA helicase HelY	Replication/repair
polyprenyl-diphosphate synthase GrcC1 [	Metabolism
folylpolyglutamate synthase FolC	Metabolism
putative aminotransferase	Metabolism
tyrosyl-tRNA synthetase TyrS	Translation
bifunctional glutamine-synthetase adenylyltransferase/deadenyltransferase	Cellular Functions
formate dehydrogenase, A chain	Metabolism
oxidoreductase	Metabolism
sulfate adenylyltransferase subunit 2	Metabolism
inosine 5-monophosphate dehydrogenase	Metabolism
hypothetical protein MAB 3083c	Translation
acyl-CoA synthetase	Metabolism
protoheme IX farnesyltransferase	Cellular Functions

hypothetical protein MAB 4507	Unknown/undefined
hypothetical protein MAB 2718c	Cellular Functions
cytochrome P450	Metabolism
short-chain Z-isoprenyl diphosphate synthetase	Metabolism
cytosol aminopeptidase	Metabolism
hypothetical protein MAB 1534	Replication/repair
phosphoribosylaminoimidazole-succinocarboxamide synthase	Metabolism
para-aminobenzoate synthase component II	Metabolism
putative Mrp-like protein	Miscellaneous
putative ethanolamine permease	Metabolism
cysteine synthase/cystathionine beta-synthase family protein	Metabolism
delta-aminolevulinic acid dehydratase	Metabolism
putative 2,3-dihydroxybiphenyl 1,2-dioxygenase or glyoxalase/bleomycin resistance protein	Metabolism
phosphoribosylamine--glycine ligase	Metabolism
sulfate ABC transporter permease CysT	Cellular Functions
3-hydroxybutyryl-CoA dehydrogenase	Metabolism
UDP-galactopyranose mutase (Glf)	Cellular Functions
peptidase U62, modulator of DNA gyrase	Miscellaneous
O-antigen/lipopolysaccharide transport integral membrane protein ABC transporter RfbD	Cellular Functions
D-3-phosphoglycerate dehydrogenase	Metabolism

Supplementary Table 4. Functional classification of the 50 bottom-ranked genes.

**Gene**	**Functional Classification**
thiosulfate sulfurtransferase	Translation
dihydroxy-acid dehydratase	Metabolism
30S ribosomal protein S13	Translation
type II citrate synthase	Cellular Functions
molecular chaperone DnaK	Cellular Functions
transcription elongation factor GreA	Unknown/undefined
50S ribosomal protein L6	Translation
DNA-binding response regulator MtrA	Cellular Functions
succinyl-CoA synthetase subunit alpha	Cellular Functions
propionyl-CoA carboxylase beta chain 6	Metabolism
hypothetical protein MAB 2888c	Unknown/undefined
acyl carrier protein	Metabolism
ATP-dependent protease ATP-binding subunit ClpX	Cellular Functions
putative CarD-like transcriptional regulator	Cellular Functions
30S ribosomal protein S4	Translation
nitrogen regulatory protein P-II	Metabolism
50S ribosomal protein L27	Translation
F0F1 ATP synthase subunit C	Cellular Functions
S-adenosyl-L-homocysteine hydrolase	Metabolism

hypothetical protein MAB 3221c	Unknown/undefined
putative FeS assembly protein SufB	Cellular Functions
isocitrate lyase (AceA)	Cellular Functions
succinate dehydrogenase flavoprotein subunit	Cellular Functions
DNA-directed RNA polymerase subunit beta	Cellular Functions
30S ribosomal protein S2	Translation
30s ribosomal protein S20	Translation
ribonucleotide-diphosphate reductase subunit beta	Metabolism
DNA-directed RNA polymerase subunit beta'	Cellular Functions
50S ribosomal protein L3	Translation
RecA protein	Replication/repair
50S ribosomal protein L16	Translation
50S ribosomal protein L24	Translation
elongation factor G	Translation
glutamine synthetase, type I (GlnA1)	Metabolism
DNA-directed RNA polymerase subunit alpha	Cellular Functions
glyceraldehyde-3-phosphate dehydrogenase, type I	Metabolism
50s ribosomal protein L21	Translation
Crp/Fnr family transriptional regulator	Cellular Functions
50S ribosomal protein L5	Translation
30S ribosomal protein S8	Translation
30S ribosomal protein S1	Translation
50S ribosomal protein L11	Translation
translation initiation factor IF-1	Translation
30S ribosomal protein S11	Translation
chaperonin GroEL	Cellular Functions
elongation factor Tu	Translation
co-chaperonin GroES	Cellular Functions
30S ribosomal protein S12	Translation
.30S ribosomal protein S10	Translation
30S ribosomal protein S19	Translation

PCR amplification of *polC, ftsZ* and *Hoa* genes

Three PCR assays were designed to amplify the DNA polymerase III alpha subunit (*polC*), 4-hydroxy-2-ketovalerate aldolase (*Hoa*) and cell division protein FtsZ (*ftsZ*) genes. Primer sequences and the expected product sizes are listed in Supplementary Table 5.

DNA from *M. abscessus* ATCC 19977 was extracted by heating the bacterium in a Middlebrook 7H9 broth suspension to 100°C for 15 minutes. The 25 µl reaction mixture for the *polC* and *Hoa* gene assays consisted of 2.5 µl of template DNA, 2 µl each of forward and reverse primers (10 µM), 12.5 µl of 1x GoTaq Green Master Mix (Promega) and 6 µl of

water. For the *ftsZ* gene amplification, 0.125 µl of DMSO(0.5%) was added to 5.875 µl of water in the reaction mixture.

The thermal cycling profile for all three PCR assays consisted of an initial heating at 94°C for 5 min (for the activation of *Taq* polymerase) followed by 35 cycles of denaturation at 94°C for 1 min, annealing at 63.7°C for 1 min and extension at 72°C for 1 min, and ending with a final extension at 72°C for 5 min.

PCR products were analyzed on a 2% w/v agarose gel, purified using QIAquick PCR Purification Kit (Qiagen) and sequenced with forward and reverse primers.

Supplementary Table 5: Primer sequences and expected product size for the *polC*, *ftsZ* and *Hoa* genes

**Gene**	**Primers**	**Product size (bp)**
DNA polymerase III alpha subunit (*polC*)	pol F - GCCTTCACCAGAGATTCCGT<br>pol R - GAGGAACCGCTCAAGGACAT	644
Hydroxy-2-ketovalerate aldolase (*MAB_626*)	ald F – ATGATGTCGCACACCATCTC<br>ald R –CCATACCGCTCACCCTGAC	497
Cell division protein FtsZ (*ftsZ)*	ftsZ F - CGAGGATGCCAAGGACGAGA<br>ftsZ R - CATCGCCGCATGAAGGGC	927

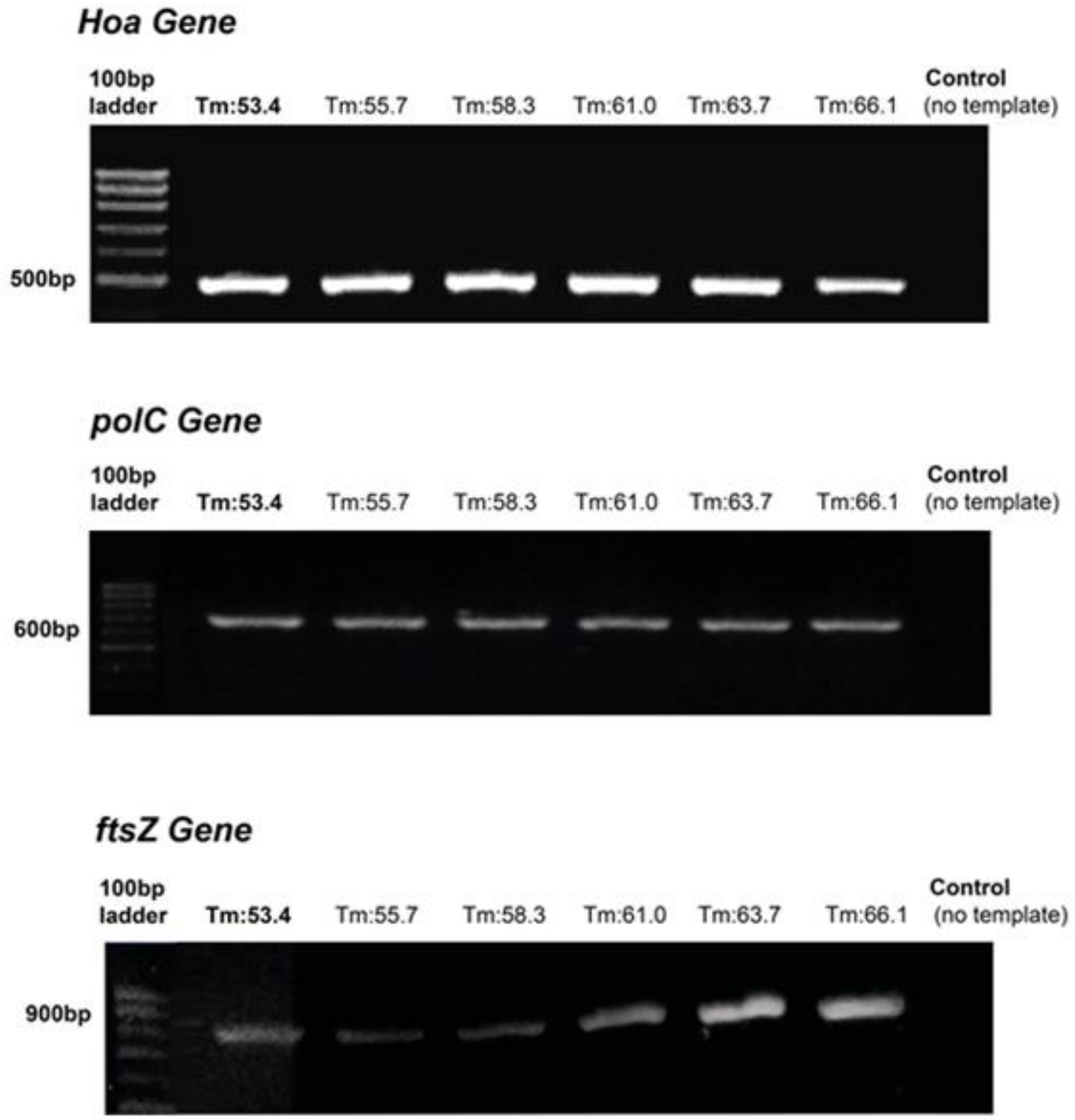

Supplementary Figure 1. Gel electrophoresis of three selected genes for the classification of *Mycobacterium abscessus* subspecies

Supplementary Table 6: NCBI accession numbers for sequences included in the study

Strain	Status	Accession
Mycobacterium abscessus ATCC 19977	Genome	NC_010397.1
Mycobacterium abscessus 3A-0119-R	Genome	NZ_AKUX00000000.1
Mycobacterium abscessus 3A-0122-R	Genome	NZ_AKUY00000000.1
Mycobacterium abscessus 3A-0122-S	Genome	NZ_AKUZ00000000.1
Mycobacterium abscessus 3A-0731	Genome	NZ_AKVA00000000.1
Mycobacterium abscessus 3A-0810-R	Genome	NZ_AKUP00000000.1
Mycobacterium abscessus 3A-0930-R	Genome	NZ_AKVB00000000.1
Mycobacterium abscessus 3A-0930-S	Genome	NZ_AKVC00000000.1
Mycobacterium abscessus 47J26	Genome	NZ_AGQU00000000.1
Mycobacterium abscessus 4S-0116-R	Genome	NZ_AKVD00000000.1
Mycobacterium abscessus 4S-0116-S	Genome	NZ_AKVE00000000.1
Mycobacterium abscessus 4S-0206	Genome	NZ_AKUT00000000.1
Mycobacterium abscessus 4S-0303	Genome	NZ_AKTU00000000.1
Mycobacterium abscessus 4S-0726-RA	Genome	NZ_AKTV00000000.1
Mycobacterium abscessus 4S-0726-RB	Genome	NZ_AKTW00000000.1
Mycobacterium abscessus 5S-0304	Genome	NZ_AKTX00000000.1
Mycobacterium abscessus 5S-0421	Genome	NZ_AKTY00000000.1
Mycobacterium abscessus 5S-0422	Genome	NZ_AKTZ00000000.1
Mycobacterium abscessus 5S-0708	Genome	NZ_AKUA00000000.1
Mycobacterium abscessus 5S-0817	Genome	NZ_AKUB00000000.1
Mycobacterium abscessus 5S-0921	Genome	NZ_AKUQ00000000.1
Mycobacterium abscessus 5S-1212	Genome	NZ_AKUC00000000.1
Mycobacterium abscessus 5S-1215	Genome	NZ_AKUD00000000.1
Mycobacterium abscessus 6G-0125-R	Genome	NZ_AKUE00000000.1
Mycobacterium abscessus 6G-0125-S	Genome	NZ_AKUF00000000.1
Mycobacterium abscessus 6G-0212	Genome	NZ_AKUR00000000.1
Mycobacterium abscessus 6G-0728-R	Genome	NZ_AKUS00000000.1
Mycobacterium abscessus 6G-0728-S	Genome	NZ_AKUG00000000.1
Mycobacterium abscessus 6G-1108	Genome	NZ_AKUH00000000.1
Mycobacterium abscessus M115	Genome	NZ_AJLZ00000000.1
Mycobacterium abscessus M139	Genome	NZ_AKVR00000000.1
Mycobacterium abscessus M148	Genome	NZ_AKVV00000000.1
Mycobacterium abscessus M152	Genome	NZ_AKVT00000000.1
Mycobacterium abscessus M154	Genome	NZ_AJMA00000000.1
Mycobacterium abscessus M156	Genome	NZ_AKVU00000000.1
Mycobacterium abscessus M159	Genome	NZ_AJSD00000000.1
Mycobacterium abscessus M172	Genome	NZ_AJSE00000000.1
Mycobacterium abscessus M24	Genome	AJLY00000000.2
Mycobacterium abscessus M93	Genome	NZ_AJGF00000000.1
Mycobacterium abscessus M94	Genome	NZ_AJGG00000000.1
Mycobacterium abscessus subsp. bolletii BD	Genome	NZ_AHAS00000000.1
Mycobacterium massiliense CCUG 48898 = JCM	Genome	NZ_AKVF00000000.1

15300		
Mycobacterium massiliense M18	Genome	NZ_AJSC00000000.1
Mycobacterium sp. JLS	Genome	CP000580.1
Mycobacterium sp. KMS	Genome	CP000518.1
Mycobacterium sp. MCS	Genome	CP000384.1

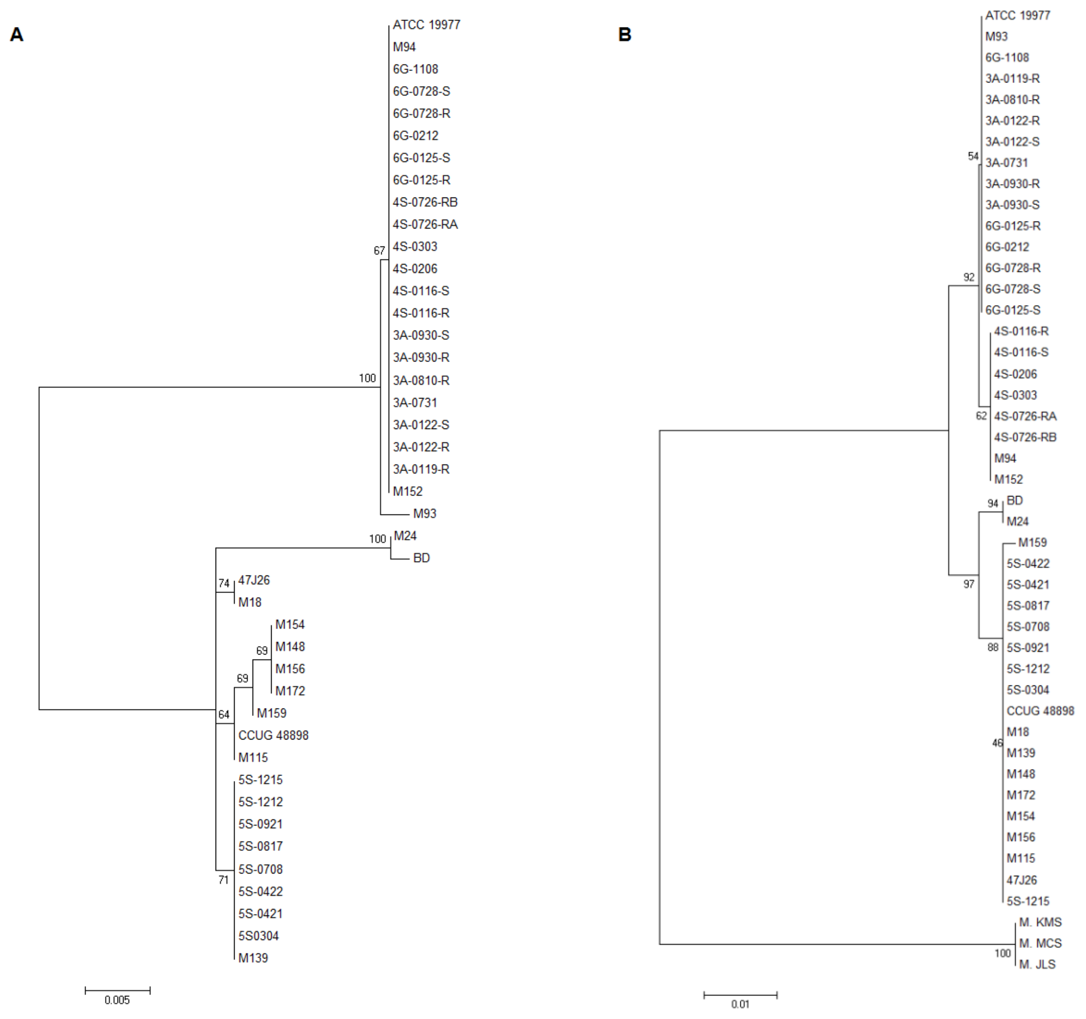

Supplementary Figure 2. Phylogenetic trees to support the minimal gene set classification. A) *rpoB*-gene based phylogeny. B) *hsp65*-gene based phylogeny. Both trees were reconstructed using MEGA 5.1 with bootstrap replication of 1000. The subspecies classification in both trees is in accordance with the phylogenomic classifications using 50 median-ranked genes as well as the 3 minimal gene set.

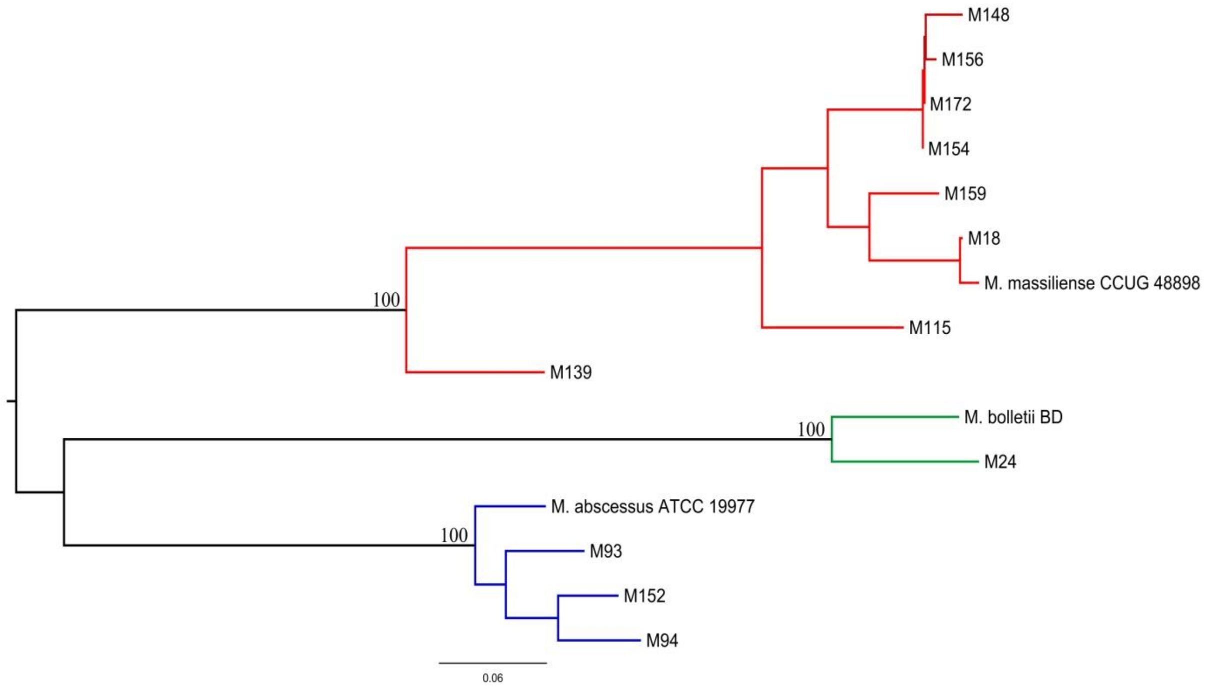

Supplementary Figure 3. SNP-based phylogenetic tree. All genome sequences were aligned using Panseq. The SNPs in the core genome (genomic regions present across all strains) were identified and retrieved. These SNPS were concatenated into a super-sequence for each strain and a phylogenetic tree was plotted using MEGA software.
